# The Human‐Like Collagen Alpha‐1 Type V Peptides Strengthen the Dermal Fiber Network and Improve the Regeneration Ability of Cells

**DOI:** 10.1111/jocd.70611

**Published:** 2025-12-21

**Authors:** Ye Hyang Kim, Byung Kuk Kim, Yeon Kyung Nam, Ha Yeon Kim, Jae Seok Lee, Eun Young Jeong, Kang Hyuk Lee, Song Seok Shin

**Affiliations:** ^1^ Life Science R&D Center Hyundai Bioland Co. Ltd. Cheongju‐si South Korea

**Keywords:** dermal fiber network, firmness, human‐like collagen alpha‐1 type V peptides, skin regeneration

## Abstract

**Background:**

Collagen, a major structural component of the skin, decreases with age and is associated with wrinkles, reduced elasticity, sagging, and dryness. While hydrolyzed or marine‐derived collagens are widely used in cosmetics, advances in biotechnology have enabled the development of bio‐collagen peptides. However, the role of collagen type V–derived peptides in skin biology remains largely unexplored.

**Aims:**

In this study, we generated human‐like collagen alpha‐1 type V peptides (hCOLVp) and investigated their effects on skin‐related parameters.

**Patients/Methods:**

COL5A1‐derived peptides with optimized sequences were generated, and a single candidate was identified through screening. Protein expression of collagen types I, III, XVII, and laminin 5 was evaluated in human dermal fibroblasts or keratinocytes. A collagen gel contraction assay and SEM‐based microstructural analysis were performed. Finally, a reconstructed human full‐thickness skin (RS) model was used to assess peptide penetration and barrier‐associated proteins.

**Results:**

hCOLVp increased expression of collagen types I, III, XVII, and laminin 5. Treated collagen gels showed greater contractile force and higher fiber density than controls. In RS models, hCOLVp penetrated over time and was associated with increased collagen fibers and upregulated expression of involucrin and transglutaminase‐1 (TGase‐1), both related to barrier function. In a preliminary clinical trial, hCOLVp improved skin barrier properties, density, and elastic recovery.

**Conclusion:**

These findings suggest that hCOLVp contributes to strengthening dermal and epidermal structural networks, thereby supporting skin function and resilience.

## 1 Introduction

The skin, the body's outermost organ, serves as an important barrier that protects against the external environment and provides resistance to repetitive mechanical stress while maintaining flexibility for movement. The extracellular matrix (ECM) is a highly organized multimolecular structure essential for the mechanical properties of the skin and for regulating cell behavior. The ECM is composed primarily of collagens, laminins, fibronectin, and elastin, with collagens being the most abundant proteins in human skin, accounting for 85%–90% of its dry weight [1]. In the adult dermis, type I collagen represents approximately 80% of total collagen, followed by type III (15%) and type V (5%) [2]. While the roles of collagen types I and III are well established, the functions of type V collagen in skin biology are less well understood [3, 4, 5].

Collagen type V, a member of the fibril‐forming collagens, regulates fibril assembly. The most abundant isoform comprises by two α1(V) chains and one α2(V) chain [6]. Observations of the role for collagen type V have demonstrated that it is critical for early fibril initiation, as well as for determining fibril number and diameter [7, 8, 9]. Generally, collagens are synthesized in cells, secreted into the ECM, and organized into striated fibrils [10, 11]. Proper assembly of the dermal matrix is essential for the skin to maintain its physiological and mechanical properties [12]. Studies have reported that collagen type V α1 (COL5A1) knockout mice synthesize normal amounts of collagen type I, but fail to form collagen fibrils and die at the onset of organogenesis [8, 13]. A representative disorder associated with collagen type V deficiency is Ehlers‐Danlos syndrome (EDS), a congenital disease that primarily affects the connective tissue of the skin and joints. Patients typically present with skin hyperextensibility delayed wound healing, and joint hypermobility [14, 15, 16]. These findings indicate that normal fibrillogenesis regulated by collagen type V is crucial for skin function.

During aging, a reduction in collagen type I, III and V has been observed in the skin [12, 17]. An increase in the expression of MMP‐1, ‐2 and ‐9 contributes to ECM degradation, including collagen fragmentation [18, 19, 20]. Age‐related thinning of collagen bundles has been reported in both the papillary and reticular dermis [21], and the aged dermis is significantly thinner than that of younger individuals [22]. These changes, including collagen degradation and reduced collagen biosynthesis, result in aberrant collagen homeostasis and net collagen deficiency [4, 23, 24, 25, 26]. Because collagen is the predominant component of the ECM, it strongly influences the activity of resident fibroblasts. In young skin, fibroblasts adhere well to intact ECM, and this adherence generates mechanical forces. In aged skin, however, ECM degradation impairs fibroblast attachment [24, 27, 28]. For these reasons, identifying agents that stimulate the production of ECM components, including collagen, remains a common anti‐aging strategy. Although age‐related collagen loss is a critical factor contributing to skin aging, restoring collagen content alone may not be sufficient to recover overall skin function. Therefore, the present study aimed to investigate whether human‐like collagen alpha‐1 type V peptides (hCOLVp) could enhance not only the synthesis of collagen and barrier‐associated proteins but also contribute to the structural organization of the dermal matrix.

## 2 Materials and Methods

### 2.1 Preparation of Human‐Like Collagen Alpha‐1 Type V Peptides

His‐tagged human COL5A1 (NCBI Gene ID: 1289) peptides were generated, with both ends cleaved using BamH1 and Xho1, and cloned into the pET‐28a (His‐tag) vector. The recombinant protein was overexpressed in the *Escherichia coli* (BL21) strain. The bacterial cells were harvested from the culture medium by centrifugation (6000× *g*, 4°C) and lysed by sonication (10 s duration with a 50 s interval) under cold condition. The lysate was centrifuged (8000× *g*, 4°C), and the supernatant was filtered through a 0.45 μm filter. Recombinant COL5A1 peptide was purified using Ni‐NTA resin (GE Healthcare, USA). Target fractions were dialyzed overnight at 4°C against PBS (pH 7.4) using 10 kDa dialysis tubing (cellulose membrane, Thermo, USA). Purified recombinant COL5A1 was visualized as a single band of approximately 20 kDa by immunoblot assay using a 6×‐His Taq antibody (Invitrogen, USA).

### 2.2 LC/MS Analysis and MS/MS Data Analysis

The SDS‐PAGE band, S1, was prepared using trypsin enzyme. All LC/MS analysis samples were analyzed with an LTQ‐Orbitrap XL (Thermo, USA) connected to an Easy‐nano LC system (Thermo, USA) incorporated with an auto‐sampler. All MS/MS samples were analyzed with MOSCOT server (Matrix science, USA).

### 2.3 Cell Culture

Normal human dermal fibroblast (NHDF, ATCC, USA), normal adult human dermal fibroblast (aNHDF, ATCC, USA) and human epidermal keratinocyte, neonatal (HEKn, Thermo, USA) were maintained in Iscove's Modified Dulbecco's Medium (IMDM, Welgene, Korea) supplemented with 10% fetal bovine serum (FBS, Gibco, USA) and 1% antibiotics (Gibco, USA) or Epilife medium supplemented with HKGS (Thermo, USA). The cells incubated at 37°C in a humidified incubator with 5% CO_2_. And the cells were then sub‐cultured with 0.25% trypsin‐0.53 mM EDTA (Gibco, USA), which was replaced with fresh medium every 2 days.

### 2.4 Cell Viability Assay

Cell viability was determined by 3‐(4,5‐dimethylthiazol)‐2,5‐diphenyl tetrazolium bromide (MTT; Sigma, USA) assay. Briefly, 2.5 mg/mL of MTT in phosphate buffered saline was added to wells of 24‐well plates and incubated at 37°C for 4 h, followed by the addition of dimethyl sulfoxide (Duksan, Korea). The absorbance was measured at 570 nm. Absorbance readings were subtracted from the value of blank wells. Cell viabilities were calculated as a percentage of control absorbance.

### 2.5 Scratch Assay

Wounds were generated using Scar Scratcher (SPL Life Sciences, Korea) at the bottom site of 24 well plate and were captured right after scratching (DMI8, Leica Camera, Germany). The medium was replaced to serum free medium and NHDFs were incubated for 24 h either with COL5A1_S1 or S2 and microscopic images were obtained from the same sites again. The open wound area of images was analyzed with Image J program. Wound closure in % is calculated by the following formula [29]:

$$\begin{array}{c} \text{closed wound area}\;\left(\%\right)=100\%\\ -\left[\left(\text{open wound area}\;24\;h/\text{open wound area}\;0\;h\right)\times 100\%\right] \end{array}$$

### 2.6 RNA Isolation, cDNA Synthesis and Quantitative Real Time‐PCR

NHDFs were treated with COL5A1_S1 or S2 and were incubated in medium without serum for 24 h. Total RNA was isolated from the cells with QIAzol (Invitrogen, USA) and then qPCRBIO cDNA synthesis kit (PCR Biosystems, USA) was used for cDNA synthesis according to the manufacturer's instructions. Using qPCRBIO SyGreen Blue Mix Lo‐ROX (PCR Biosystems, USA) following the manufacturer's protocol, qRT‐PCR reactions were performed in triplicates. Primers for the amplification of collagen type III (F: 5′‐CTG ATG GGG TCA AAT GAA GGT G‐3′, R: 5′‐CGT GCA ACC ATC CTC CAG AAC‐3′) and b‐actin (F: 5′‐GGC ACC CAG CAC AAT GAA G‐3′, R: 5′‐CCG ATC CAC ACG GAG TAC TTG‐3′) were purchased from Cosmogene Tech (Korea).

### 2.7 Immunoblotting Analysis

The culture medium was collected after incubation, and the proteins were concentrated using Amicon Ultra‐4 Centrifugal Filter Unit (MERK, USA). Cells were lysed with Pro‐prep protein extraction solution (iNtRON, Korea), and the lysates were centrifuged at 4°C. The supernatant was collected into fresh tubes. For immunoblotting, proteins were separated on 7% NUPAGE Tris‐acetate gels or 8% Bis‐Tris gels and transferred to PVDF membranes using iBlot2 Dry Blotting system (Invitrogen, USA). The membranes were incubated overnight at 4°C with antibodies against collagen type I (Abcam, USA), collagen type III (Santa cruz, USA), laminin 5 (Santa cruz, USA), collagen type XVII (Thermo, USA), β‐actin (Santa cruz, USA), and GAPDH (Santa cruz, USA). After washing, the membranes were incubated with horseradish peroxidase‐conjugated secondary antibodies (Bio‐Rad, USA) at room temperature for 1–2 h. Blots were developed using Westsave Gold (AbFrontier, Korea) according to the manufacturer's instructions. Protein levels were analyzed using Luminograph II and CSAnalyzer4 software (Atto, Japan).

### 2.8 Collagen Gel Contraction Assay and Analysis of Gel's Properties

Collagen gels were made using rat‐tail type I collagen (Sigma, USA). All materials were kept on ice to avoid premature collagen polymerization. A collagen gel solution was made containing 1‐part 10× DMEM (Sigma, USA), 8‐parts collagen with sterile water, 1N NaOH and 1‐part cell suspension. The solution was dispensed into 12 well plate (Thermo, USA) and polymerized for 30 min in an incubator at 37°C. Next, 1 mL medium containing 10% FBS and hCOLVp was added to each well and incubated for further polymerization at 37°C. For floating model, gels were dislodged from the bottom of the plates using a sterile spatula after incubation for 90 min and stayed in the incubator by next day. hCOLVp was treated again and incubated for 24 h. The contraction of collagen gels was analyzed by taking pictures and measuring the diameter using Image J program. The microstructure of the collagen gel drying with hexamethyldisilizane (HMDS) and was confirmed using scanning electron microscopy (SEM, Hitachi) [25, 30]. The mechanical property of collagen gel was analyzed by measuring hardness using Rheometer (Compac‐100, Sun Scientific, Japan).

### 2.9 Masson's Trichrome and Immunofluorescence Staining on RS

The RS, KeraSkin‐FT, was purchased from Biosolution in Korea. To evaluate the effects of hCOLVp on collagen fiber and skin barrier related proteins. After treatment for 24 h, the RS was collected and fixed in 10% neutral buffered formalin and was dehydrated according to the sequential process of the tissue processor (Excelsior ES, Thermo, USA). And then, histological analysis was processed. Paraffin sections (5 μm) were stained with hematoxylin–eosin (H&E), Masson's trichrome and immune‐fluorescent antibodies. To conduct the immunofluorescence analysis, the sections were incubated in antigen retrieval solution (Thermo, USA) and then treated with peroxidase suppressor solution (Thermo, USA) for 30 min. Next, the sections were incubated with blocking solution (Thermo, USA) for 30 min and reacted with primary antibodies, involucrin (Santa Cruz, USA) and TGase‐1 (Santa Cruz, USA) for 24 h. The slides were incubated in secondary antibody, Mouse Alexa Flour 488 (Thermo, USA) for 1 h. After washing with 1× PBS, they were mounted with VECRASHIELD Vibrance Antifade Mounting Medium with DAPI (Vector laboratories, USA). The histologic characteristics, the collagen fibers and the expression of proteins were observed under microscope (DMI8, Leica, Germany).

### 2.10 Skin Absorption of hCOLVp in RS

Topical skin absorption of hCOLVp was evaluated using the RS, KeraSkin‐FT. The labeling process was carried out according to the manufacturer's method (Alexa Fluor 488 Microscale Protein Labeling Kit, Thermo, USA). Briefly, dye‐conjugated and purified hCOLVp was applied on the RS for 6 and 18 h, and fixed in 10% neutral buffered formalin. Subsequently, tissue samples were washed with DW, immersed in 10% and 20% sucrose solution for 4 h each, and then transferred to 30% sucrose solution. We used a freezing microtome (Leica, Germany) to produce 15 μm‐thick cryo‐sections, which were then imaged using a fluorescence microscope (DMI8, Leica Camera, Germany).

### 2.11 Clinical Trials

This study was a double‐blinded and placebo‐controlled clinical trial. And it was performed at skin research center between October 23 and November 29, 2023. While the design was not randomized, the split‐arm or split‐periocular region methodology reduced inter‐individual variability. A total of 23 participants were recruited over a one‐week period, from which 20 eligible subjects were enrolled (Table 1); all were healthy adult aged 20–60 years and had no skin disease or skin allergies. The test was carried out under constant temperature and humidity (20°C–24°C, 40%–60%) without air movement and direct sunlight.

**TABLE 1:** CONSORT participant flow.

Phase	Description	*n*
Enrollment	Assessed for eligibility	23
	Excluded	3
	Not meeting inclusion criteria	3
	Declined to participate	0
Allocation	Randomized/Allocated	20
	Test (Right forearm: hCOLVp cream)	20
	Control (Left forearm: Vehicle cream)	20
Follow‐up	Lost to follow‐up	0
	Discontinued	0
Analysis	Completed and analyzed (per protocol)	20

*Note:* A total of 23 individuals were assessed for eligibility, and 3 were excluded. Twenty participants received either hCOLVp cream or vehicle cream as provided by the clinical institution. All participants completed the study and were included in the final analysis.

In this study, the formulation containing hCOLVp was assigned to the test group, whereas the vehicle formulation without the active ingredient was assigned to the placebo‐control group. To ensure blinding, the investigational products were coded and provided to the clinical institution as “Cream A” and “Cream B,” without disclosure of their contents to either the investigators or participants.

- Skin elasticity restoration: The test or vehicle cream was applied once in a dosage of 2 μL/cm^2^ to the test sites (forearm), and changes in skin depressions volume due to skin pressure were compared and evaluated.
- Skin density: The test or vehicle cream was applied topically to the designated facial area (periocular region) twice daily for 4 weeks. Clinical evaluations were performed at baseline (week 0), after 2 weeks, and after 4 weeks of product application.
- Skin barrier function: The test or vehicle cream was applied topically to the designated forearms twice daily for 4 weeks. Clinical evaluations were performed at baseline (week 0), after 2 weeks, and after 4 weeks of product application.

### 2.12 Participants

Twenty healthy female volunteers (mean age 41.3 ± 11.3 years; range 22–60) were enrolled. Inclusion and exclusion criteria are as follows:

#### 2.12.1 Inclusion Criteria

- Adults between 20 and 60 years of age,
- Individuals who fully understood the purpose and procedures of the study and voluntarily signed a written informed consent form prior to participation,
- Absence of dermatological or systemic diseases,
- Not pregnant or lactating,
- No recent medical or cosmetic procedures on the application sites,
- For trials assessing skin barrier function, individuals with objectively confirmed impairment, defined as TEWL ≥ 12.0 g/m^2^·h at the designated test site,
- Individuals who were available for follow‐up assessments throughout the study period.

#### 2.12.2 Exclusion Criteria

- Declined participation or failed to provide written informed consent,
- Pregnant, lactating, or planning pregnancy within the next 6 months,
- History of topical corticosteroid use for longer than 1 month within the past 6 months,
- Presence of sensitive or hypersensitivity skin,
- Skin abnormalities at the test site (e.g., nevi, acne, erythema, or telangiectasia),
- Cosmetic or dermatologic procedures performed on the test site within the past 6 months,
- Presence of psychiatric disorders or intellectual disabilities.

### 2.13 Skin Elasticity Restoration

Skin elasticity restoration was assessed using Antera 3D (Miravex Limited, Ireland), a three‐dimensional skin imaging device. The designated test area was photographed at baseline (before product application) and immediately after a single application of the test product. The captured images were analyzed with the manufacturer‐provided software to calculate the volume (mm^3^) of depressions. A decrease in depression volume indicated a reduction in skin pressure marks, which was interpreted as an improvement in skin elasticity restoration. All measurements were expressed in cubic millimeters (mm^3^).

### 2.14 Dermal Density

Skin density was evaluated using Ultrascan UC22 (C&K electronic, Germany) at baseline (week 0), after 2 weeks, and after 4 weeks of product application. After image acquisition, the captured cross‐sectional images were analyzed using the manufacturer's proprietary software. The primary outcome variable for this analysis was density (%), with higher values indicating improved skin density.

### 2.15 Barrier Function Assessment

Skin barrier function was assessed by measuring transepidermal water loss (TEWL) using a Tewameter TM 300 (C&K Electronic, Germany) at baseline, 2 weeks, and 4 weeks. TEWL values were expressed in g/m^2^·h, with lower values indicating improved barrier function.

### 2.16 Statistical Analysis

Data were analyzed using the statistical analysis program SPSS statistics. Statistical analyses were performed using both *p*‐values and effect sizes with 95% confidence intervals, in order to evaluate not only the presence of statistically significant differences but also the magnitude and reliability of the observed effects. The effect sizes for each clinical evaluation item were calculated and recorded in Table 2.

- Within‐group comparisons (pre‐ vs. post‐application): *the Paired samples t‐test* was used in the case of parametric analysis, and *the Wilcoxon signed rank test* was used for non‐parametric analysis.
- Between‐group comparisons (test group variance vs. control group variance): *the Independent samples t‐test* was used in the case of parametric analysis, and *the Mann–Whitney U test* was used for non‐parametric analysis.

**TABLE 2:** Effect sizes (*dz*) and statistical analysis of time‐dependent improvements.

Outcome	Comparison	Mean change	SD	Cohen's *dz*	*t*‐value	*p*	95% CI
Elasticity restoration	Test ref‐single use	−10.38	3.31	−3.14	−14.02	< 0.001	[−11.9, −8.9]
	∆ Test‐control	−5.69	3.50	−1.60	−5.8	< 0.001	[−7.8, −3.6]
Skin density	Test ref‐2 weeks	+0.60	2.70	+0.22	+0.99	n.s.	[−0.66, 1.86]
	Test ref‐4 weeks	+3.00	2.70	+1.11	+4.97	< 0.001	[1.74, 4.26]
	Control ref‐2 weeks	+0.50	2.40	+0.21	+0.93	n.s.	[−0.62, 1.62]
	Control ref‐4 weeks	+0.30	2.20	+0.14	+0.61	n.s.	[−0.73, 1.33]
TEWL	Test ref‐2 weeks	−2.73	1.96	−1.39	−6.23	< 0.001	[−3.65, −1.82]
	Test ref‐4 weeks	−5.72	2.45	−2.34	−10.45	< 0.001	[−6.86, −4.57]
	Control ref‐2 weeks	−0.86	2.35	−0.37	−1.64	n.s.	[−1.96, 0.24]
	Control ref‐4 weeks	−2.24	2.59	−0.87	−3.88	< 0.01	[−3.45, −1.04]

*Note:* Mean change, SD, effect size (Cohen's *dz*), *t*‐value, *p*‐value, and 95% CI are summarized for elasticity restoration, skin density, and TEWL at single use, 2 weeks, and 4 weeks. Significant improvements were observed in the test group compared with controls, particularly in elasticity restoration (single use, 4 weeks), skin density (4 weeks), and TEWL (2 and 4 weeks).

## 3 Results

### 3.1 Identification and Effects of Recombinant COL5A1 Proteins

**FIGURE 1:**
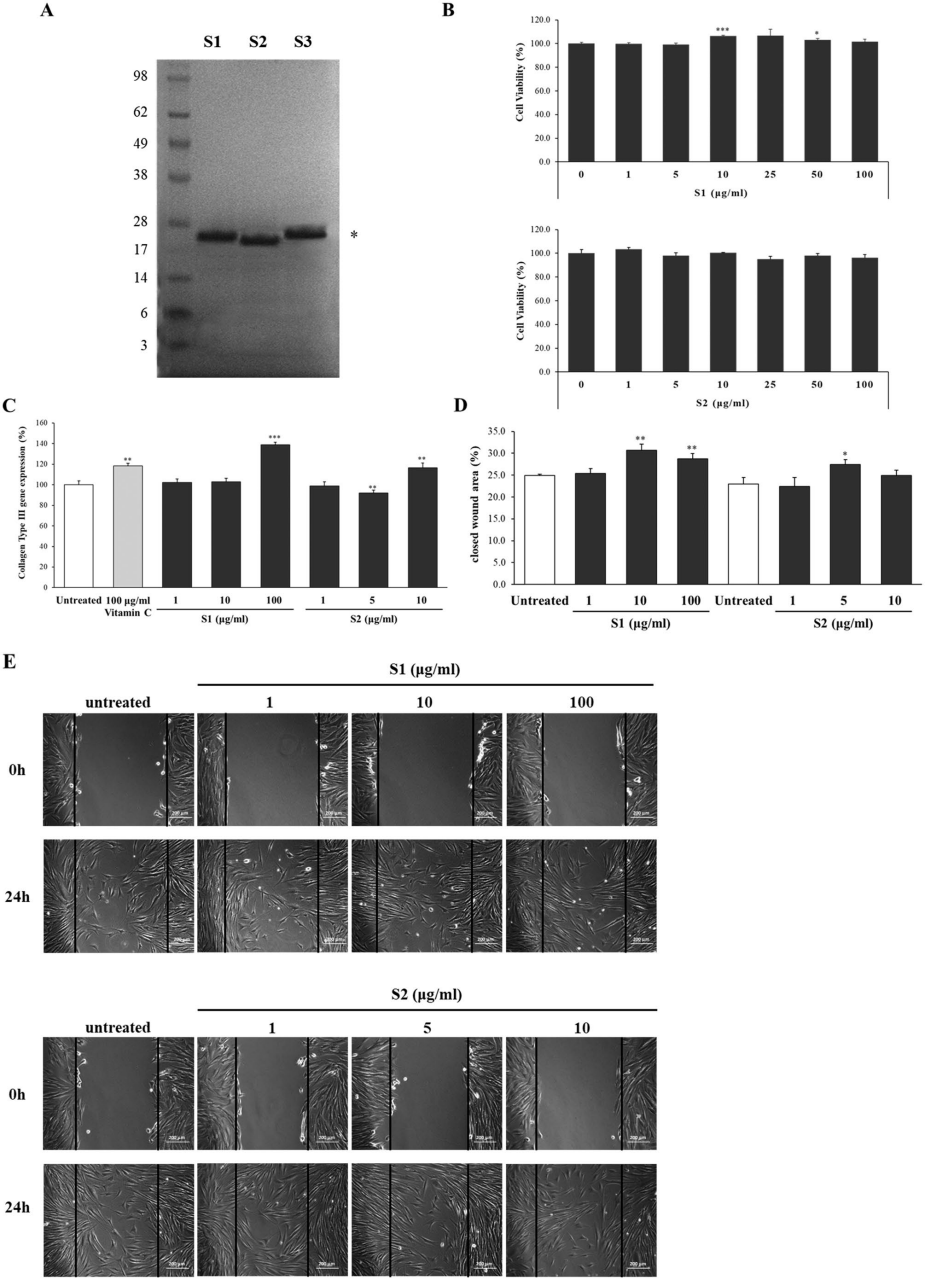
Identification of three human COL5A1 peptide candidates and comparative evaluation of the cellular effects of two peptides. (A) SDS‐PAGE analysis demonstrated approximate molecular weights of 22 kDa for S1, 20 kDa for S2, and 23 kDa for S3. Each peptide was purified to a final purity exceeding 95%. (B) The cytotoxicity of S1 and S2 in NHDFs exposed to increasing concentrations (0–100 μg/mL) for 24 h was assessed using an ELISA microplate reader at 570 nm. (C) Total RNA isolated from NHDFs treated with S1 or S2 was analyzed by quantitative PCR. Collagen type III mRNA expression was normalized to β‐Actin. Data are presented as mean ± SD from three independent experiments. *p < 0.05, **p < 0.01, ***p < 0.001. (D, E) NHDFs treated with S1 or S2 were subjected to an in vitro scratch assay. Wound closure was monitored over a 24 h period, and wound margins were quantified using Image J program. *p < 0.05, **p < 0.01.

Proline‐rich peptides are crucial in collagen synthesis and help stimulate fibroblasts to produce more collagen. Additionally, these peptides are known for their hydrophilic and anti‐oxidant properties that help effectively retain moisture and protect the skin [31]. In this study, we choose three peptide sequences with a high content of proline in COL5A1 (NCBI Gene ID: 1289, UniProt accession number: P20908), S1 sequence (901–1020), S2 sequence (1321–1440), S3 sequence (1521–1640) (Table S1). For cloning, optimized sequences corresponding to each peptide were synthesized. They were cloned into pET‐28a (His‐taq) vector and recombinant proteins were overexpressed in the *E. coli* strain. To confirm their molecular weight, we conducted SDS‐PAGE, and the peptide bands were analyzed using an anti‐His antibody. Three recombinant peptides had single bands and the molecular weight of S1, S2 and S3 were found to be about 22, 20, and 23 kDa respectively (Figure 1A).

Next, we tested the effects of two peptides on cells for selecting the best candidate, excluding S3, which has low protein expression. To evaluate the cytotoxicity of S1 and S2, samples were treated at various concentrations in NHDFs, and MTT assay was performed. S1 showed no cytotoxicity at concentrations between 0 and 100 μg/mL. In S2, cells exhibited morphological alterations at concentrations ranging from 25 to 100 μg/mL (Figure 1B).

COL5A1 S1 and S2 increased collagen type III gene expression at the last concentration, 36.8% (*p* < 0.001) and 16.6% (*p* < 0.01), respectively (Figure 1C). But, their two peptides did not have an effect on collagen type I gene expression (data was not shown). And S1 and S2 decreased wound margin by 30.7% (*p* < 0.01) and 27.4% (*p* < 0.05) after scratching on fibroblast cells (Figure 1D,E). Based on these findings, S1, which demonstrated greater induction of collagen type III gene expression and enhanced cell migration, was selected and subsequently designated as hCOLVp.

**FIGURE 2:**
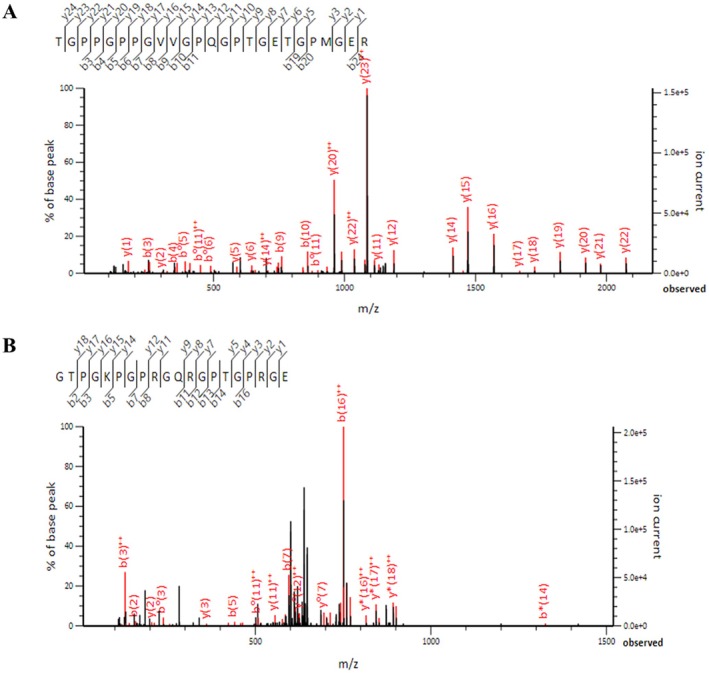
Identification of hCOLVp by LC–MS/MS analysis. The MS/MS spectra confirmed the presence of the peptide TGPPGPPGVVGPQGPTGETGPMGER (1181.059 *m*/*z*) generated by trypsin digestion (A), and the peptide GTPGKPGPRGQRGPTGPRGE (654.014 *m*/*z*) generated by Glu‐C digestion (B).

And we analyzed the S1 peptides by liquid chromatography‐mass spectrometry (LC/MS). Figure 2 and Table S2 show a representative MS/MS spectrum for the identified peptides, which originated from S1 (554 TGPPGPPGVVGPQGPTGETGPMGER 573, 1181.059 *m*/*z*) and (465 GTPGKPGPRGQRGPTGPRGE, 654.014 *m*/*z*).

### 3.2 hCOLVp Stimulates the Production of Collagen Types and DEJ Components

**FIGURE 3:**
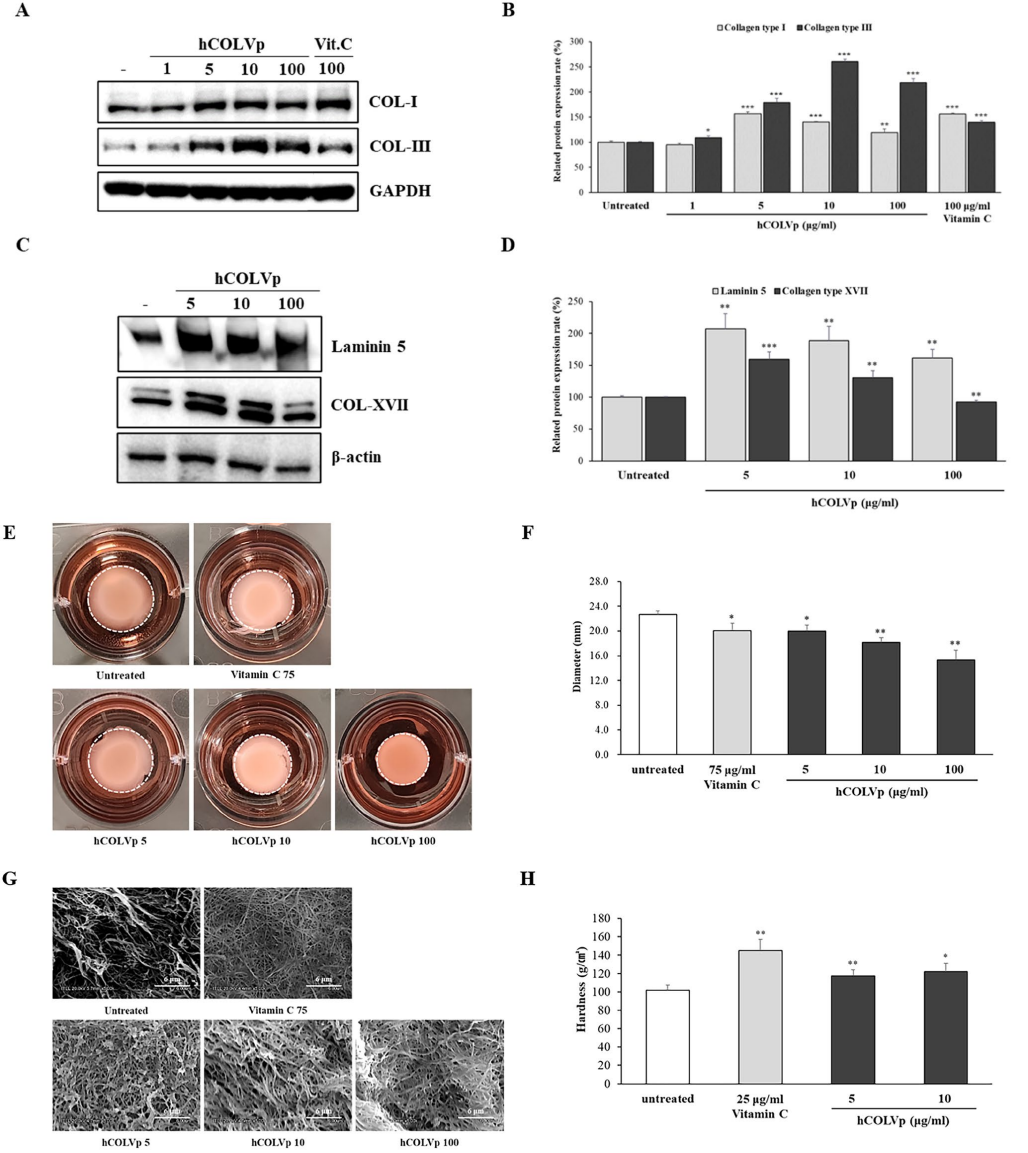
Effects of hCOLVp on the protein expression of collagen types and laminin 5, and on the dermal matrix environment. Total protein was isolated from dermal fibroblasts and A431 cells treated with hCOLVp for 24 h. Immunoblot analyses were performed using antibodies against collagen type I, III (A, B), and collagen type XVII and laminin 5 (C, D). Protein expression levels were normalized to GAPDH or β‐Actin. Data are presented as mean ± SD from three independent experiments. *p < 0.05; **p < 0.01; ***p < 0.001. Fibroblast‐mediated collagen gel contraction is shown in (E), and gel diameters were quantified using Image J program (F). The microstructure of the collagen gels was examined by scanning electron microscopy (SEM) (G) (scale bar = 6.0 μm). The textural properties of the collagen gels were assessed using a rheometer (table speed: 100 mm/min; hold 0.2 mm) (H). *p < 0.05; **p < 0.01.

To investigate the effect of hCOLVp on cutaneous composition, we measured the expression of collagen type I and III, which are the predominant forms in the dermis. As shown in Figure 3A,B, treatment with hCOLVp, increased collagen type I and III protein levels by approximately 57.1% and 160.3%, respectively (*p* < 0.001). Immunoblotting further demonstrated that hCOLVp upregulated the expression of laminin 5 by 107.3% (*p* < 0.01), and collagen type XVII by 59.4% (*p* < 0.001) (Figure 3C,D). These results indicate that hCOLVp modifies ECM composition and is also associated with changes in epidermal junction proteins.

### 3.3 hCOLVp Affects the Formation of Collagen Fiber Network

The collagen gel contraction assay (CGCA) is a method to visualize cell‐ECM mechanical interactions. ECM play an essential role in regulating the physiological activities of resident cells, including adhesion, proliferation, migration, differentiation, and maintaining normal homeostasis [32]. And the properties of aged dermal fibroblast are reduced mobility and plasticity [33]. aNHDF embedded in collagen gels and treated with hCOLVp exhibited significantly greater contractility than untreated cells (Figure 3E). In the absence of hCOLVp, gel diameter was 22.67 mm, whereas hCOLVp treatment reduced the diameter to 15.33 mm, corresponding to a 32.4% increase in contractility (Figure 3F). Next, we investigated whether hCOLVp could affect the properties of collagen gel. Increased fiber density was observed in hCOLVp‐treated gels by SEM (Figure 3G), and gel hardness was elevated by 23.6% (Figure 3H). These findings indicate that hCOLVp alters ECM properties in a biomimetic environment.

### 3.4 hCOLVp Is Absorbed in RS and Promotes the Formation of Skin Barrier and Dermal Fibers

**FIGURE 4:**
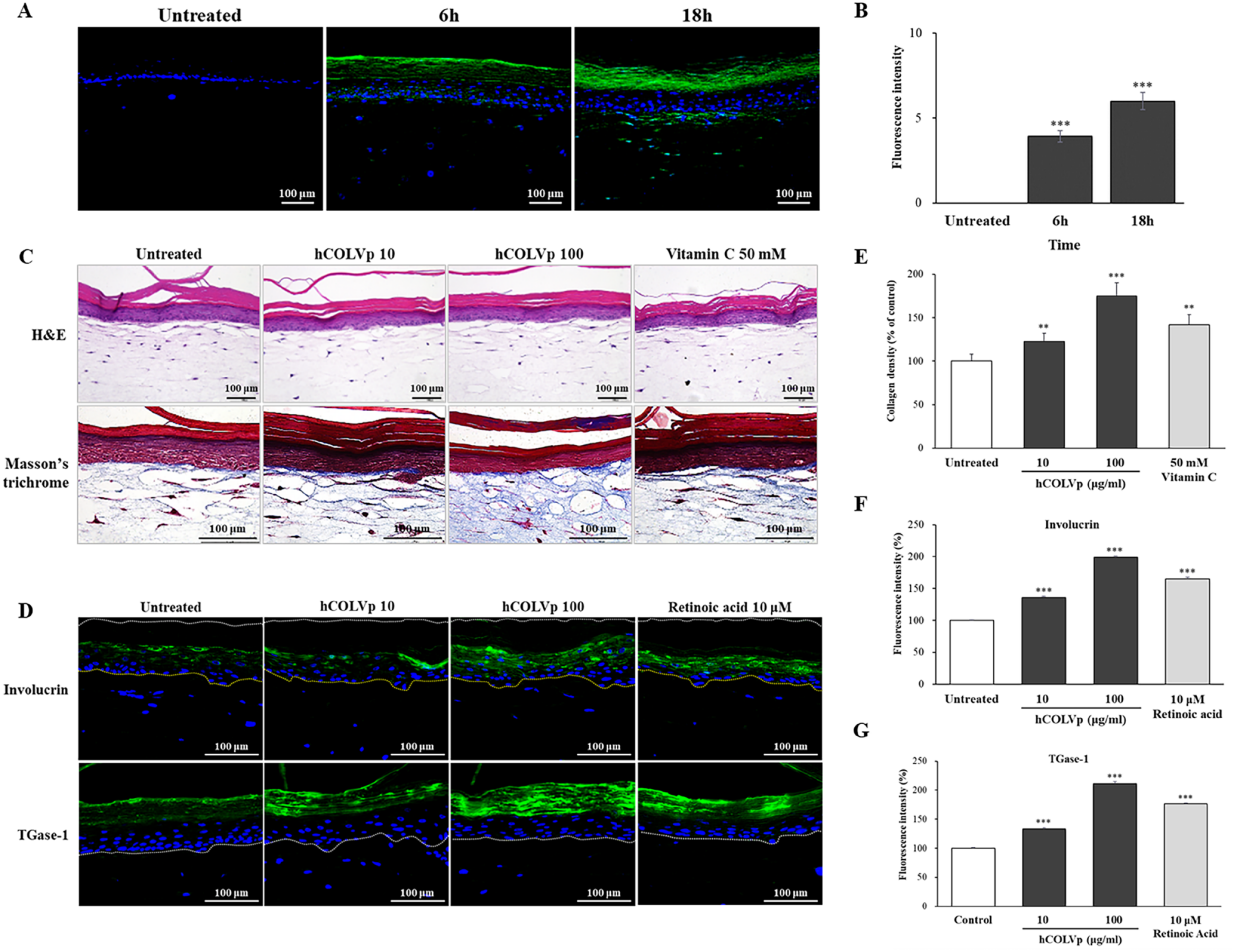
Visualization of hCOLVp absorption and the staining of collagen and skin barrier markers in the RS model. Images of sections treated with hCOLVp labeled with fluorescence dye were taken using immunofluorescence staining (A). Fluorescence intensity over time was analyzed using Image J program (B). Statistical significance for panel B was evaluated for time‐dependent changes, and significance levels are denoted as *p < 0.001. The collage fiber density was visualized in blue using Masson's trichrome staining (C). Immunofluorescence staining was performed using antibodies raised against involucrin and TGase‐1 (D). Green fluorescence indicates their expression. The staining area was quantified and analyzed using the Image J program (E–G). Scale bar = 100 μm. Values represent the mean ± SD of three independent measurements. *p < 0.05; **p < 0.01; ***p < 0.001.

In a further study, we investigated the extent of hCOLVp absorption into RS. After 6 h of treatment, hCOLVp penetrated through the stratum corneum into the basal layer, and after 18 h it was detected in the upper dermis (Figure 4A). Fluorescence intensity increased to 5.99 at 18 h compared with control (Figure 4B). To assess the impact of hCOLVp from dermis to epidermis under three‐dimensional conditions, RS models were used. In the hCOLVp group, collagen fiber density increased by 175.2% relative to control (Figure 4C,E). The expression of involucrin and TGase‐1 was also significantly elevated, by 198.9% and 211.1%, respectively, compared with control (Figure 4D,F,G). From these results, hCOLVp was found to influence the organization of dermal collagen fibers and was associated with increased expression of skin barrier‐related proteins.

### 3.5 hCOLVp Improves Skin Elasticity Restoration, Density and Barrier Function

**FIGURE 5:**
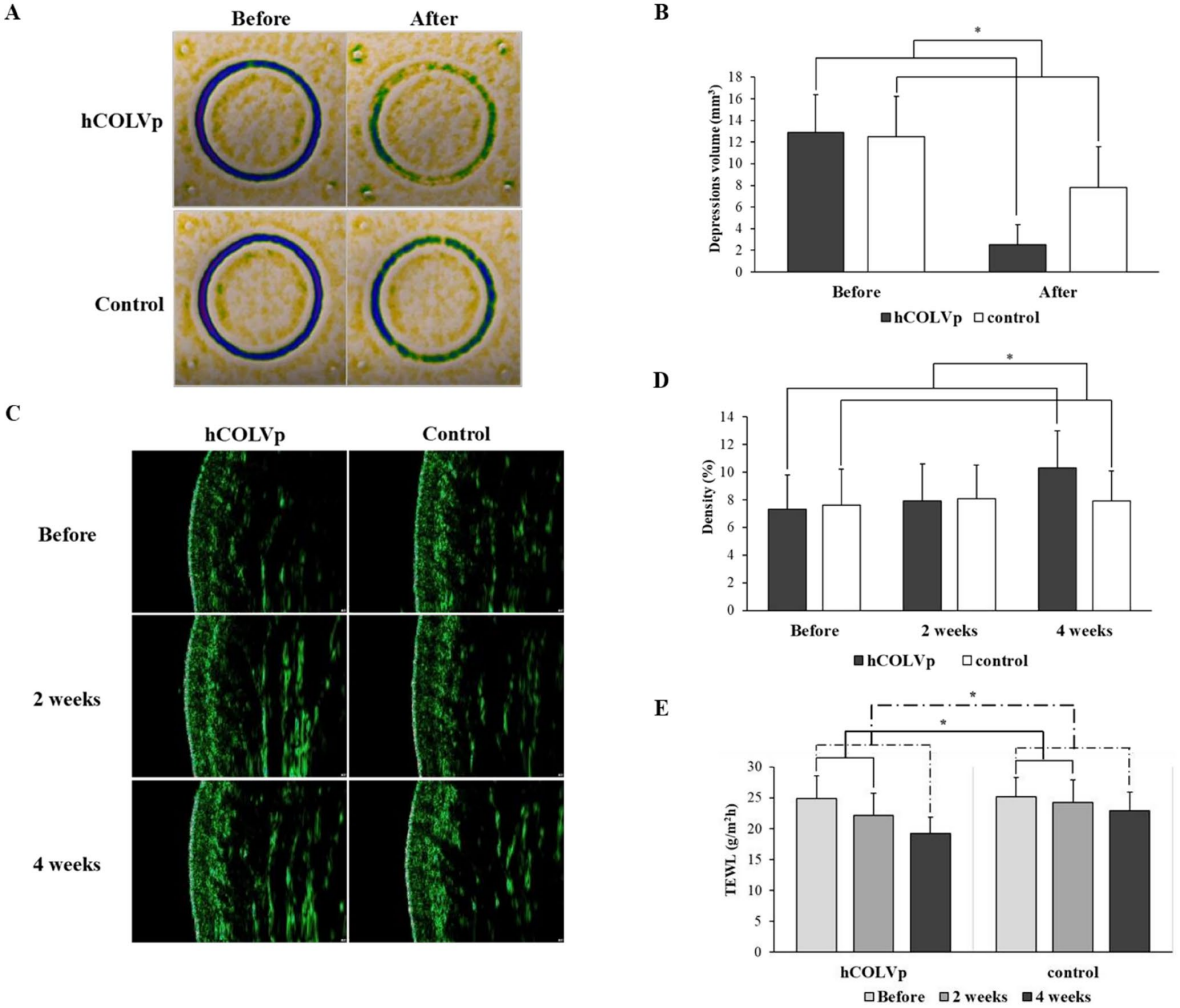
Changes in skin condition following topical application of hCOLVp. Representative images illustrating skin elasticity before and after application of the test product are shown in (A, B). Dermal density (C, D) and TEWL (E) were evaluated after 4 weeks of treatment with either placebo‐control or hCOLVp. Data are presented as mean ± SEM. *p < 0.05 versus baseline.

As shown in Figure 5A,B, skin elasticity restoration measured 12.900 mm^3^ before using the product and 2.517 mm^3^ after using hCOLVp once. The control group was 12.524 mm^3^ before using and 7.833 mm^3^ after using the product. In Table 2, statistical significance was confirmed in hCOLVp group compared to the control group. And the improvement rate after using hCOLVp was 80.8% (*p* < 0.001). After application of hCOLVp, skin density was 7.9% after 2 weeks and 10.3% after 4 weeks of using the product (Figure 5C,D). In Table 2, a considerable increase was shown at from baseline to 4 week (+3.00 ± 2.70; *p* < 0.001) and the improvement rate after 4 weeks was 47.3%. However, after 2 weeks, there was no substantial change. The control group showed no meaningful changes at both 2 and 4 weeks. And next, skin barrier measured 24.926 g/m^2^h before using hCOLVp, and after 2 and 4 weeks was 22.193 g/m^2^h, 19.208 g/m^2^h respectively (Figure 5E). The control group was 25.147 g/m^2^h before using the product, 24.288 g/m^2^h after 2 weeks and 22.902 g/m^2^h after 4 weeks. And the improvement rate after 2 weeks was 10.6%, and after 4 weeks was 22.5%. The hCOLVp‐treated group showed a notable decrease from baseline to both 2 and 4 weeks (*p* < 0.001), while the control group showed a slight decrease only at 4 weeks (*p* < 0.01) (Table 2). These results suggest that hCOLVp has a significant clinical effect in improving skin elasticity, density, and barrier function.

## 4 Conclusion

In this study, hCOLVp was found to stimulate collagen synthesis in fibroblasts and promote fibrillogenesis in dermal collagen gels. In reconstructed human full‐thickness skin models, it was associated with improvements in parameters related to barrier function, collagen fiber density, and dermal structure. Although the clinical outcomes did not achieve full statistical significance, the findings provide preliminary evidence that hCOLVp may contribute to dermal matrix organization, barrier integrity, and skin mechanical properties.

## 5 Discussion

The ECM is a highly organized structure that plays a key role in the mechanical properties of the skin and in regulating cell behavior [3, 4]. Most ECM components are collagens, and it is important to increase or supplement collagen content, which decreases with age [4, 22, 34]. However, maintaining collagen levels alone may not be sufficient for proper collagen function. Fibrillogenesis, the process by which newly synthesized collagen molecules are assembled into fibrils, is essential for conferring strength, firmness and elasticity to the dermis [9, 10, 35]. Through this process, the dermal matrix acquires its physiological and mechanical characteristics [12]. Collagen type V, although comprising only about 5% of the dermis, plays a critical role in initiating fibrillogenesis and regulates fibril number and diameter [7, 8, 9]. With aging, reduction in collagen type I, III, and V, together with increased MMPs activity, contribute to collagen degradation and impaired biosynthesis [12, 17, 18, 19, 20]. These changes result in abnormal fibrillar networks [25, 26], dermal thining [21, 22] and loss of structural and functional properties of the dermal matrix [24, 27, 28].

We considered collagen fibril formation, rather than a simple increase in ECM components, to be essential for the mechanical and physical functions of the skin. Therefore, collagen type V, which regulates fibril assembly and determines fibril number and diameter [7, 8, 9], was selected as the focus of this study. Because full‐length peptides are poorly absorbed through the skin, we generated partial‐sequence peptides derived from human collagen alpha‐1 type V and evaluated their effects on the skin. Treatment with hCOLVp increased the production of collagen types I and III (Figure 3A,B). To assess its impact on fibrillogenesis, we used a dermal mimetic collagen gel containing aNHDFs. Gels treated with hCOLVp showed greater contractile force and increased fiber density compared with untreated gel, as confirmed by SEM microstructural analysis (Figure 3E–H). Upregulation of collagen fibers was also observed in RS models (Figure 4C,E). Given that aged skin characterized by reduced ECM components and impaired fibroblast‐ECM interactions [24, 27, 28], these findings suggest that hCOLVp may promote fibroblast‐ECM interactions and support matrix formation.

In addition, hCOLVp was detected from the basal layer to the upper dermis after 18 h, indicating transdermal penetration over time (Figure 4A,B). In normal epidermal keratinocytes, hCOLVp induced the expression of laminin 5 and collagen type XVII (Figure 3C,D), while the barrier‐related proteins involucrin and TGase‐1 were upregulated in RS (Figure 4D,F,G). Collectively, these results suggest that hCOLVp affects the dermis, dermal‐epidermal junction, and epidermis, thereby contributing to skin structural support. Furthermore, preliminary clinical trials indicated improvements in skin elasticity restoration, density, and barrier function (Figure 5, Table 2).

Our findings strongly suggest that hCOLVp contributes to dermal matrix reorganization and skin barrier improvement. To minimize variability and reduce potential confounding factors in the clinical outcomes, the study was conducted under a standardized measurement environment with the application of a region‐splitting method. However, improvements in clinical parameters may be partially attributable to the intrinsic moisturizing properties of the formulation vehicle, or uncontrolled lifestyle and environmental factors among participants. This study was conducted with 20 participants, which is a typical sample size for exploratory cosmetic clinical trials and thus relatively small. Similar studies have been reported, including one involving 22 Asian women that evaluated the effects of collagen tripeptide on skin wrinkles, elasticity, density, and the accumulation of advanced glycation end‐products [36], and another in which a second cohort of 20 participants was assessed for various parameters such as skin hydration, brightness, and wrinkle reduction following the use of an anti‐aging cream [37]. To substantiate the observed benefits of hCOLVp, additional independent studies with larger, more diverse cohorts, longer follow‐up, and head‐to‐head comparisons with other collagen‐based interventions are required.

Most collagen peptides used in cosmetics are derived from animals or marine sources, and they mainly act by supplying amino acids to support collagen synthesis [38, 39, 40]. While these peptides may provide moisturizing and skin barrier benefits, their absorption efficiency and bioactivity are limited. In contrast to such conventional peptides, recent research has focused on the biological activity of type I collagen‐derived dipeptides and tripeptides. Pro‐Hyp, for example, has been reported to stimulate fibroblast growth and extracellular matrix production [41], and it also plays a role as a signaling molecule during wound healing [42]. And collagen tripeptides have demonstrated beneficial effects such as improving skin elasticity and density, as well as reducing advanced glycation end‐products (AGEs) [36]. Such active components, including retinoids and hyaluronic acid, commonly used in dermocosmetics, play important roles in collagen formation, inhibition of degradation, and regeneration [43, 44, 45, 46].

In this context, hCOLVp was found to directly regulate fibrillogenesis of dermal collagen type V fibers. Unlike conventional collagen peptides, hCOLVp goes beyond simply supplying amino acids and instead supports the structural organization and functional improvement of dermal collagen. In this regard, hCOLVp represents a novel dermocosmetic peptide with potential applications in improving skin elasticity and barrier function. In combination with active agents such as retinoids and vitamins, hCOLVp may reinforce synthesis‐related signaling pathways and thereby improve their functional efficacy. Beyond its role in preventing age‐related collagen degradation, hCOLVp may also improve fibrillar structural integrity and mitigate qualitative changes in collagen, supporting and potentially enhancing the activity of established cosmetic ingredients.

Taken together, these mechanistic insights are supported by our experimental findings. In this study, hCOLVp stimulated collagen synthesis in fibroblasts, promote fibrillogenesis in dermal collagen gels, and regulate barrier‐associated proteins in reconstructed skin models. When it considered alongside the clinical observations, these results suggest that hCOLVp may contribute to dermal matrix organization and exert beneficial effects on skin elasticity and density.

## 6 Limitations

This exploratory cosmetic trial was conducted with a limited sample size (*n* = 20) and without adjustment for multiple comparisons, which restricts the generalizability of the findings. Moreover, the short observation period (2–4 weeks) does not permit a comprehensive evaluation of long‐term efficacy and safety, including potential adverse effects such as fibrosis. Although a double‐blinded, placebo‐controlled design was employed to minimize bias, these methodological constraints should be acknowledged. Future studies should include larger and more diverse populations, incorporate formal power calculations, and extend follow‐up periods to validate and expand upon the present findings.

## 7 Potential Bias

All authors of this study are affiliated with Hyundai Bioland Co. Ltd., which developed and provided the investigated peptides. Such affiliation may potentially introduce bias in study design, data interpretation, and reporting. To minimize these risks, the basic research, evaluation, and material preparation were divided and conducted independently by each department, following standardized experimental protocols. The clinical trial was carried out at an independent institution, reviewed and approved by the Institutional Review Board (IRB), and clinical assessments were performed using validated instruments.

## Author Contributions

Conceptualization, Y.H.K. and Y.K.N.; methodology, formal analysis, investigation, Y.H.K., B.K.K., Y.K.N. and H.Y.K.; resources, B.K.K. J.S.L. and K.H.L.; writing – original draft preparation, project administration Y.H.K.; supervision, writing – review and editing, E.Y.J. and S.S.S. All authors have read and agreed to the published version of the manuscript.

## Funding

The authors have nothing to report.

## Ethics Statement

The clinical trial was conducted at ProbeM Skin Research Center (Daejeon, Republic of Korea) from October 23 to November 29, 2023. Ethical approval was obtained from the Institutional Review Board (IRB Nos. GCC‐038‐23‐001, GCC‐027‐23‐001 and GCC‐055‐23‐001; approval date: December 8, 2023). The study followed the principles of the Declaration of Helsinki and adhered to Good Clinical Practice (GCP) standards.

## Consent

All participants provided written informed consent prior to participation.

## Conflicts of Interest

The authors declare no conflicts of interest.

## Supporting information


**TABLE S1:** The amino acid sequences of three COL5A1 peptide candidates. Three peptide sequences derived from the COL5A1 protein were designed and synthesized as candidate molecules. Each sequence represents a distinct partial fragment of the human COL5A1 chain selected for further screening.
**TABLE S2:** Identification peptides of hCOLVp by peptide mass fingerprinting analysis. Peptide mass fingerprinting analysis was performed to confirm the identity of the synthesized hCOLVp. The table lists the detected peptide fragments, their observed mass/charge (*m*/*z*) values, theoretical values, and sequence matches, demonstrating consistency with the expected COL5A1‐derived peptide sequence.

## Data Availability

Data supporting the findings of this study are available from the corresponding author upon reasonable request.
