# Supplementary material for: The Human‐Like Collagen Alpha‐1 Type V Peptides Strengthen the Dermal Fiber Network and Improve the Regeneration Ability of Cells
Source: J Cosmet Dermatol. 2025 Dec 21;24(12):e70611. doi: 10.1111/jocd.70611 (PMC12719547; doi:10.1111/jocd.70611)
Supplement: Supplementary file 1 — TABLE S1: The amino acid sequences of three COL5A1 peptide candidates. Three peptide sequences derived from the COL5A1 protein were designed and synthesized as candidate molecules. Each sequence represents a distinct partial fragment of the human COL5A1 chain selected for further screening. TABLE S2: Identification peptides of hCOLVp by peptide mass fingerprinting analysis. Peptide mass fingerprinting analysis was performed to confirm the identity of the synthesized hCOLVp. The table lists the detected peptide fragments, their observed mass/charge (m/z) values, theoretical values, and sequence matches, demonstrating consistency with the expected COL5A1‐derived peptide sequence. [file JOCD-24-e70611-s001.docx]

Supplementary Table 1. The amino acid sequences of three COL5A1 peptide candidates.

| Candidates of COL5A1 peptides | Site of gene | Sequences |
| --- | --- | --- |
| S1 | 901-1020 | GGATCCATGGTPGKPGPRGQRGPTGPRGERGPRGITGKPGPKGNSGGDGPAGPPGERGPNGPQGPTGFPGPKGPPGPPGKDGLPGHPGQRGETGFQGKTGPPGPPGVVGPQGPTGETGPMGERGHPGPPCTCGAG |
| S2 | 1321-1440 | GGATCCATGGPPGPKGPPGDDGPKGSPGPVGFPGDPGPPGEPGPAGQDGPPGDKGDDGEPGQTGSPGPTGEPGPSGPPGKRGPPGPAGPEGRQGEKGAKGEAGLEGPPGKTGPIGPQGAPGKPGPDGLRCTCGAG |
| S3 | 1521-1640 | GGATCCATGTGPSGPIGPPGPPGLPGPPGPKGAKGSSGPTGPKGEAGHPGPPGPPGPPGEVIQPLPIQASRTRRNIDASQLLDDGNGENYVDYADGMEEIFGSLNSLKLEIEQMKRPLGTQQNPARTCKCTCGAG |

Supplementary Table 2. Identification peptides of hCOLVp by peptide mass fingerprinting analysis.

| Enzyme | m/z | Amino acid sequence | # of match | Score | Charge | # Missed Cleavages |
| --- | --- | --- | --- | --- | --- | --- |
| Trypsin | 1181.059 | TGPPGPPGVVGPQGPTGETGPMGER | 22 | 116.77 | 2.10E-12 | Oxidation (M); Oxidation (P) |
|  | 704.3555 | GPNGPQGPTGFPGPK | 9 | 114.52 | 3.50E-12 |  |
|  | 861.449 | GPPGPPGKDGLPGHPGQR | 17 | 102.07 | 6.20E-11 |  |
|  | 662.7971 | GNSGGDGPAGPPGER | 8 | 99.17 | 1.20E-10 |  |
|  | 699.6989 | GPNGPQGPTGFPGPKGPPGPPGK | 21 | 79.92 | 1.00E-08 |  |
|  | 911.0911 | GNSGGDGPAGPPGERGPNGPQGPTGFPGPK | 16 | 73.55 | 4.40E-08 | Oxidation (P) |
|  | 726.6938 | GITGKPGPKGNSGGDGPAGPPGER | 11 | 65.57 | 2.80E-07 | Oxidation (P) |
|  | 778.1427 | GPNGPQGPTGFPGPKGPPGPPGKDGLPGHPGQR | 20 | 64.35 | 3.70E-07 |  |
|  | 1050.4948 | GETGFQGKTGPPGPPGVVGPQGPTGETGPMGER | 13 | 64.09 | 3.90E-07 | Oxidation (M) |
|  | 517.2609 | DGLPGHPGQR | 8 | 53.02 | 5.00E-06 |  |
|  | 887.9391 | GITGKPGPKGNSGGDGPAGPPGERGPNGPQGPTGFPGPK | 28 | 51.63 | 6.90E-06 |  |
|  | 505.8592 | GPPGPPGKDGLPGHPGQRGETGFQGK | 11 | 50.66 | 8.60E-06 |  |
|  | 1045.7448 | DGLPGHPGQRGETGFQGKTGPPGPPGVVGPQGPTGETGPMGER | 22 | 48.61 | 1.40E-05 | Oxidation (M); Oxidation (P) |
|  | 433.7455 | GTPGKPGPR | 5 | 34.16 | 0.00038 |  |
|  | 1134.8807 | GNSGGDGPAGPPGERGPNGPQGPTGFPGPKGPPGPPGK | 22 | 32.66 | 0.00054 |  |
|  | 353.6975 | GPPGPPGK | 4 | 30.68 | 0.00086 |  |
|  | 388.8982 | GPRGITGKPGPK | 14 | 26.72 | 0.0021 |  |
|  | 626.3166 | GPRGITGKPGPKGNSGGDGPAGPPGER | 19 | 21.09 | 0.0078 | 2 Oxidation (P) |
|  | 412.1998 | GETGFQGK | 7 | 18.94 | 0.013 |  |
|  | 919.9512 | DGLPGHPGQRGETGFQGK | 11 | 17.87 | 0.016 |  |
|  | 974.1349 | TGPPGPPGVVGPQGPTGETGPMGERGHPGPP | 5 | 13.26 | 0.047 | Oxidation (M);  2 Oxidation (P) |
| Glu-C | 603.3212 | RGPRGITGKPGPKGNSGGD | 13 | 38.52 | 0.00014 |  |
|  | 654.014 | GTPGKPGPRGQRGPTGPRGE | 12 | 14.04 | 0.039 |  |
